# Influenza Virus Type A Serosurvey in Cats

**DOI:** 10.3201/eid1304.060736

**Published:** 2007-04

**Authors:** Paltrinieri Saverio, Spagnolo Valentina, Giordano Alessia, Moreno Martin Ana, Luppi Andrea

**Affiliations:** * University of Milan, Milan, Italy; † Istituto Zooprofilattico Sperimentale della Lombardia e dell’Emilia, Brescia, Italy

**Keywords:** type A orthomyxovirus, influenza, cat, letter

**To the Editor**: Recent reports of cats positive for H5N1 type A influenza virus (*1*) raised the hypothesis that cats might have an epidemiologic role in this disease. Experimental findings seem to support this hypothesis. Experimentally infected cats might act as aberrant hosts (as do humans and other mammals), with symptoms and lesions developing and the virus subsequently spreading to other cats (*2*,*3*). The experimental conditions under which this occurs, however, can rarely be observed for domestic or wild cats. No spontaneous cases of transmission from cat to cat or cat to mammal have been reported, and scientifically validated reports about spontaneous disease in cats are rare (*4*–*6*). Reports about cats with circulating influenza virus antibodies are even more rare and occur in unusual epidemiologic situations (*7*). The true susceptibility of cats to type A influenza viruses in field conditions thus remains to be elucidated.

Based on the assumption that partially susceptible animals should mount an antibody response, we investigated the possible presence of antibodies against the nucleocapsid protein A (NPA), a common antigen of type A influenza viruses, expressed by both avian and human strains (*8*), in feline serum samples stored at the University of Milan and collected from 1999 to 2005. Only samples for which complete information regarding the cat (owned vs. free-roaming) and its health status were included in the study. Cats were grouped as healthy or sick on the basis of clinical signs; a complete clinicopathologic screening that included routine hematologic tests, clinical biochemical tests, and serum protein electrophoresis; serologic tests for feline immunodeficiency virus and feline leukemia virus infection, which are known to induce immunosuppression; and information regarding the follow-up, including postmortem examination for dead animals. Specifically, 196 serum samples satisfied the inclusion criteria in terms of anamnestic information about the sampled cat and, according to the above-mentioned diagnostic approach, cats were grouped as reported in the Table. Owned cats were mainly living in the urban area of Milan. By contrast, approximately half of the free-roaming cats included came from rescue shelters from a rural area northwest of Milan. Sixty samples (58.8%) from owned cats and 51 samples (54.2%) from free-roaming cats were collected from September to February, when seasonal human influenza peaks.

**Table:** Survey of feline serum samples, collected from 1999 to 2005, for influenza A virus, Italy*

Clinical status	N	Diagnosis	N	Virus status	N
Pet cats					
Nonsymptomatic	25			FIV	2
				FeLV	1
Symptomatic	77	FIP	36		
		Locally extensive inflammation	18	FIV	4
		Hematologic neoplasia	8	FIV	1
		Nonhematologic tumors	8		
		Systemic inflammatory or degenerative diseases	7	FIV	2
				FIV + FeLV	1
Free-roaming cats					
Nonsymptomatic	54			FIV	5
Symptomatic	40	Locally extensive inflammation	27	FIV	7
		Systemic inflammatory or degenerative diseases	7		
		FIP	6		

*FIV, feline immunodeficiency virus; FIP, feline infectious peritonitis; FeLV, feline leukemia virus.

Serologic tests for antibodies to type A influenza virus were performed with a competitive ELISA to detect NPA antibodies (*9*). Negative control serum from specific-pathogen-free chickens and positive control serum specimens from different species (avian, swine, and equine) were included in each plate to provide a full range of controls. Serum samples were considered positive when the absorbance value was reduced to at least 75% compared with 100% for negative control wells.

All cats were negative for type A influenza virus antibodies. The ELISA we used has been validated in several species, including humans (*9*). Antibodies against NPA are not a major response to influenza infection but likely would have been detected if infections of cat were widespread. Thus, although no positive feline serum samples were used as positive controls, the negative results are not likely false negatives. Indeed, the negative results of many cats included in the study (the free-roaming ones, especially those affected by severe illness, for which a natural cat/flu virus interaction is unrealistic) might be due to low exposure to the virus because avian influenza outbreaks never occurred in the sampling area included in this study (*10*). By contrast, many owned cats (those sampled during the winter) likely were exposed to human type A influenza viruses, since approximately half of the viruses responsible for human seasonal influenza isolated in Europe, especially in Italy, are type A (*8*,*10*). The close contact between pets and their owners probably exposed cats to these viruses; nevertheless, none of the pet cats seroconverted, even when they had severe systemic diseases or viral induced immunosuppression. Although the number of cats in this study might be statistically insufficient to show low seroprevalences, our results further support the hypothesis that, in field conditions, cats are most probably not susceptible to type A influenza viruses, especially to the human ones (e.g., H3N2, the most diffused among humans, which also did not induced symptoms or lesions in experimental conditions [*2*]) circulating in the “pre-cat flu era.” In futures studies, these results can be used to compare the results of seroepidemiologic investigations among cats living in sites contaminated by avian viruses.
